# Impact of mobility restrictions on the dynamics of transmission of COVID-19 in Colombian cities

**DOI:** 10.1093/inthealth/ihab064

**Published:** 2021-10-07

**Authors:** Angel Paternina-Caicedo, Nelson Alvis-Guzmán, Carmelo Dueñas, Javier Narvaez, Adrian D Smith, Fernando De la Hoz-Restrepo

**Affiliations:** Universidad del Sinú, Cartagena, Colombia; Universidad de Cartagena, Cartagena, Colombia; Universidad de la Costa, Barranquilla, Colombia; Universidad de Cartagena, Cartagena, Colombia; Universidad El Bosque, Bogotá, Colombia; University of Oxford, Oxford, UK; Universidad Nacional de Colombia, Bogotá, Colombia

**Keywords:** cases, COVID-19, non-pharmacological interventions, transmission dynamics

## Abstract

**Background:**

Our aim was to study the association between case rates and reductions in urban mobility in state capitals of Colombia.

**Methods:**

We designed an ecological time-series study to correlate the Colombian incidence rate with reductions in mobility trends of retail stores.

**Results:**

The meta-analysis of β coefficients describing the association between case rates and reductions in mobility trends of retail stores resulted in a mean estimate of 0.0637 (95% confidence interval 0.027 to 0.101; p<0.001) with nearly 100% heterogeneity.

**Conclusions:**

We recommend continuing to consider mobility restrictions when the number of cases starts to climb in each local jurisdiction.

## Introduction

The coronavirus disease 2019 (COVID-19) pandemic was first detected in Wuhan, China in late 2019 and efforts to exert transmission control using non-pharmacological interventions (NPIs) have disrupted lives worldwide. The potential of NPIs to curtail the pandemic case rates has been studied in multiple countries in Europe.^1^ These studies provide evidence of the impact of NPIs on sudden acute respiratory syndrome coronavirus 2 (SARS-CoV-2) transmission dynamics at the national level. However, the use of aggregated data on NPIs may result in ecological bias when assessing the efficacy of restrictive public health measures for the reduction of SARS-CoV-2 dynamics in each country. In particular, local mobility restrictions are a source of heterogeneity in COVID-19 transmission dynamics across subnational areas that can be hidden when aggregating data across an entire country. Moreover, mobility can be a highly sensitive but underutilized indicator of interpersonal contact.

Colombia implemented wide restrictions at the city level to reduce COVID-19 transmission. In the present study we looked at the association between COVID-19 case rates and reductions in urban mobility in local settings in Colombia.

## Methods

We designed an ecological time-series study to correlate incidence rates due to COVID-19^2,3^ with reductions in mobility trends of retail stores in major Colombian state capital cities. Mobility data were obtained from the COVID-19 Community Mobility Reports, collected and published by Google.^4^ The analysed period was 27 February 2020–31 January 2021. These reports include six indexes of mobility (related to retail commerce, grocery shopping, parks, transit, work and home) and estimate the mobility reduction compared with a baseline period (3 January–6 February 2020). We chose the retail mobility as the closer proxy of the reduction in contacts resulting from local lockdowns in each city. All other indicators are either related to excepted types of mobility (groceries, home, work or transit) or are considered leisure activities (parks). Missing data were imputed using Kalman filtering and state-space analyses.

We used two methodological approaches to explore the association between mobility reduction and COVID-19. First, we fitted autoregressive integrated moving average (ARIMA) models with regressors, using the daily case rates of COVID-19 in each city as the dependent time series (lagged 7 d to account for the incubation period)^5^ and the reduction in retail mobility as a continuous regressor. We then meta-analysed the results of the ARIMA coefficients across cities and reported the summary estimator along with the p-value and heterogeneity (Supplementary Figure 1, panel A). As a sensitivity analysis to account for the possible effect of holidays on mobility, for the sake of comparison we estimated the same models using only the data from 3 January to 6 February 2021 (Supplementary Figure 2).

Second, we used a t-test to compare the average weekly case rates, contrasting those periods with and without a high reduction in retail-related mobility (reductions of 25%, 50% or 75%, data lagged for 1 week)^5^ (Supplementary Figure 1, panel B). The supplementary material expands on our methods. We reported all rates per 100 000 population and p-values <0.05 were considered statistically significant.

## Results

**Figure 1.**
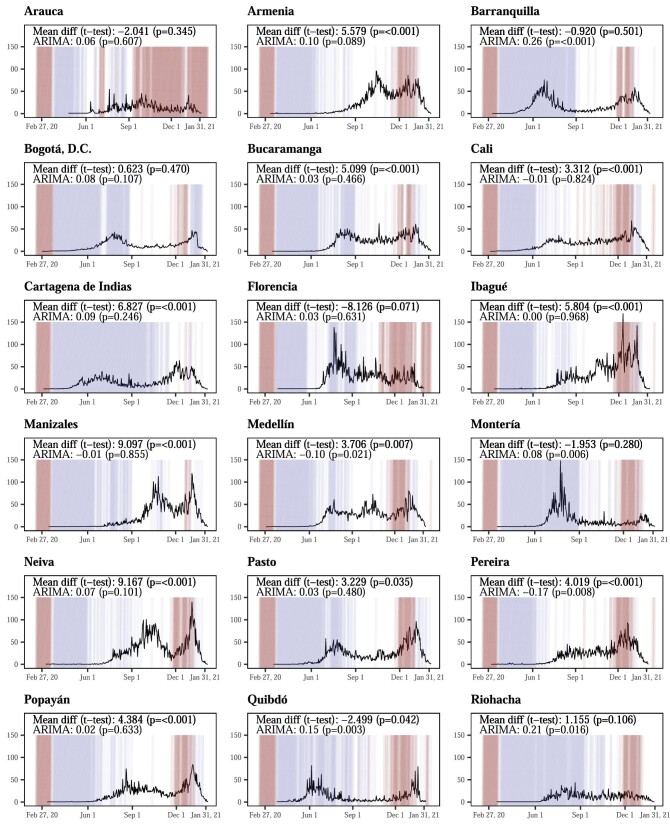

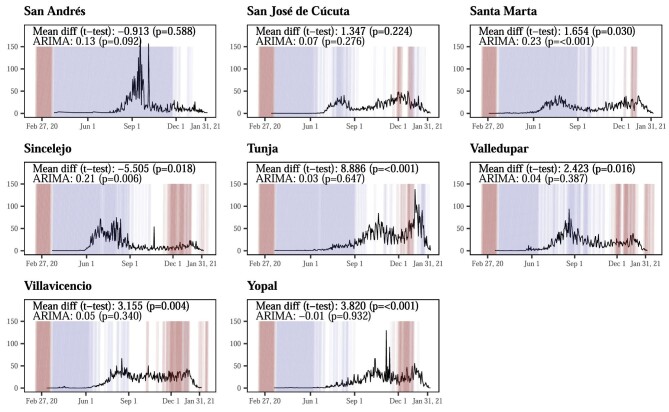
Time trends of symptomatic case rates (per 100 000 population) in 26 cities of Colombia, along with reductions >50% (blue) and <25% (red) in mobility from retail stores* and p-value of association between changes in mobility trends of retail stores using ARIMA and t-test (<50% reductions of mobility). Mobility data were extracted daily from Google Community Mobility trends.^4^ Significant negative mean differences signal that the reductions of cases were greater in time periods with lower restrictions of mobility in retail stores.

A total of 26 Colombian state capitals had 1 215 416 cases and 35 745 deaths due to COVID-19 between 27 February 2020 and 31 January 2021, for a crude mortality rate of 157.7 deaths per 100 000 and a case fatality rate of 2.9%. The city with the largest crude mortality rate was Villavicencio, with 259.4 deaths per 100 000 (see Figure 1 and Supplementary Table 1).

β coefficients for the association between changes in mobility and case rate derived from the ARIMA models ranged between −0.18 (Pereira) and 0.26 (Barranquilla). The meta-analysis of β coefficients describing the association between incidence case rates and change in mobility trends of retail stores resulted in a mean estimate of 0.0637 (95% confidence interval [CI] 0.027 to 0.101; p<0.001), with heterogeneity close to 100% (Supplementary Figure 1, panel A). This positive correlation indicates that case rates decreased after mobility in retail stores was reduced. The sensitivity analyses accounting for the effect of holidays did not reach statistical significance.

The t-tests comparing COVID-19 case rates in Colombian cities during periods of high vs low mobility reduction offered similar results. Only two cities, Quibdó and Sincelejo, showed significantly higher case rates with less mobility in the complete-case analyses when using a cut-off above vs below a 50% reduction in mobility) (Supplementary Figure 1, panel B). All other significant associations with case rates, at all pre-specified cut-off values (mobility reductions of 25%, 50% and 75%), showed fewer cases with less retail-related mobility. Overall, 15 of 65 associations (76%) were statistically significant (Supplementary Table 2). The significance of the t-test did not change after imputation (Supplementary Table 3).

## Discussion

Our work adds to the increasing body of evidence on the impact of restrictions of mobility and NPI to reduce the transmission of SARS-CoV-2 in jurisdictions worldwide. We found large heterogeneity in our results, signalling the disparities in restrictions, income, norms and local culture in each Colombian city. In several countries, such as Colombia, restrictions on mobility are being applied at the local level, therefore more granular analyses might be more appropriate than the country-wide ecological analyses currently in the literature.

We recommend, based on this work, to continue to consider mobility restrictions when the number of cases starts to climb in a local jurisdiction. However, in addition to COVID-19 cases, vaccination coverage, mental health, education and other concerns related to well-being should also be considered before starting mobility restrictions.

## Supplementary Material

ihab064_Supplemental_File

## Data Availability

All data are publicly available. Code and data for this analysis are available upon reasonable request.
