# Supplementary material for: Impact of mobility restrictions on the dynamics of transmission of COVID-19 in Colombian cities
Source: Int Health. 2021 Oct 7;14(3):332–5. doi: 10.1093/inthealth/ihab064 (PMC9070454; doi:10.1093/inthealth/ihab064)

Supplementary Methods

Design and sources

We designed and carried out an ecological study in 26 state capitals of Colombia with more than 40,000 inhabitants and available data on retail-related mobility. We used individual-level data about Covid-19 symptomatic cases, collected and anonymously published in a public repository (1) by the Colombian National Institute of Health (Instituto Nacional de Salud, *INS*). Population data for each Colombian state capital was obtained from the governmental statistical agency (Departamento Administrativo Nacional de Estadísticas, *DANE*)(2). These two pieces of information were combined to produce COVID-19 case rates at the city level, according to date of start of symptoms.

The mobility data was collected from the "COVID-19 Community Mobility Reports" of Google, available for each selected city in Colombia (3), from February 15th of 2020 to January 31st of 2021. This mobility data includes six indicators of change in mobility from baseline (retail, grocery, parks, transit, work, and home). According to Google, the baseline period went from January 3rd to February 6th, 2020. We selected the change in retail mobility from baseline for our analyses. For analytical purposes, the continuous change in mobility relative to baseline was categorized using cutoff values of 25, 50 and 75% reduction in mobility. In Google Mobility reports, Cartagena Province, and Cucuta, referred to Cartagena de Indias and San Jose de Cucuta, respectively.

We analyzed the available data comprised between February 27th of 2020 and January 31st of 2021. We excluded three capital cities because they did not have enough available mobility data (missing data >50%: Mocoa, San Jose del Guaviare and Leticia), and three capitals with less than 40,000 people (Inírida, Mitú, and Puerto Carreño).

Data analyses

We fitted a set of ARIMA models with errors (ARIMA) to Covid-19 case rates for each city, including change in retail mobility as an external regressor (lagged to 7 days (4)), and reported the P-value of the coefficient at the city level. We additionally meta-analyzed the beta coefficients and standard errors of the ARIMAX estimations using random effects models. We performed a sensitivity analysis of the model with ARIMA regression, only including Covid-19 symptomatic case rates from January 3rd to February 6th, 2021. This sensitivity analysis was fitted in order to account for changes in mobility patterns during the official holiday season in the country. Missing data was imputed using time-series imputation, with Kalman Filtering and state-space models (5).

After fitting the models using the 'auto.arima' function, we visually inspected the residuals autocorrelation function, and used the Box-Ljung residuals test to assess model fit. In our data, no ARIMA model fitted with the 'auto.arima' function resulted in a significant Box-

Ljung residual test, suggesting a good overall fit for all the evaluated models. While we occasionally observed isolated lagged residual autocorrelations slightly out of the 95% confidence intervals (**Supplementary Figure 4**), we leaned towards the most parsimonious, yet informative, model as indicated by the 'auto.arima' optimization approach.

We also used a t test to compare the weekly case rates (lagged for 1 week before the start of symptoms) contrasting periods below and above different cutoff values for reduction of retail stores-related mobility(cutoffs of 25%, 50%, or 75%). The case rates were adjusted by the number of weeks that were taken during each cutoff period (both below and above the cutoff). These weights would allow to compare rates with different time lengths.

The meta-analysis assumed the coefficients of the ARIMA with error model were continuous, and then meta-analyzed the raw means of these coefficients. The heterogeneity tests were estimated using standard formulae.

We used an automatic procedure in R (v4.0.3, built in the function 'auto.arima' of the package 'forecast') to select between alternative ARIMA models. For the meta-analysis, we used the 'metafor' package. The package 'imputeTS' was used for time-series imputation.

References

1. Instituto Nacional de Salud. Instituto Nacional de Salud | Colombia Bienvenido (a) [Internet]. 2020 [cited 2021 Feb 20]. Available from: http://www.ins.gov.co/Paginas/Inicio.aspx

2. Population and Demography [Internet]. [cited 2021 Feb 20]. Available from: https://www.dane.gov.co/index.php/en/statistics-by-topic-1/population-and-demography

3. Google. Informes de Movilidad Local sobre el COVID-19 [Internet]. 2020 [cited 2021 Feb 20]. p. 1. Available from: https://www.google.com/covid19/mobility/

4. Wei WE, Li Z, Chiew CJ, Yong SE, Toh MP, Lee VJ. Morbidity and Mortality Weekly Report Presymptomatic Transmission of SARS-CoV-2-Singapore.

5. Moritz S, Bartz-Beielstein T. imputeTS: Time Series Missing Value Imputation in R.

Supplementary Table 1. Population and crude Covid-19 mortality rates in major state capitals of Colombia.

City	Population	Crude mortality rate
Arauca	96,814	76.4
Armenia	304,764	192.6
Barranquilla	1,274,250	167.1
Bogotá, D.C.	7,743,955	164.7
Bucaramanga	607,428	204.6
Cali	2,252,616	155.2
Cartagena de Indias	1,028,736	92.2
Florencia	173,011	253.2
Ibagué	541,101	201.4
Manizales	446,160	99.7
Medellín	2,533,424	128.5
Montería	505,334	171.8
Neiva	364,408	238.2
Pasto	392,589	193.3
Pereira	477,027	143.4
Popayán	325,477	92.5
Quibdó	130,825	82.6
Riohacha	201,839	93.1
San Andrés	57,433	71.4
San José de Cúcuta	777,106	223.8
Santa Marta	538,612	114
Sincelejo	293,951	147.3
Tunja	179,263	87
Valledupar	532,956	111.8
Villavicencio	545,302	115.7
Yopal	177,688	73.7

* Between February 15th of 2020 and January 31st of 2021.

Supplementary Table 2. Differences in symptomatic Covid-19 case rates during the time with reductions of mobility trends in retail stores, at different cutoff values (25%, 50% and 75%) of weekly mobility reduction, in the complete-case analysis.

City	Reduction >75%		Reduction>50%		Reduction >25%	
	**Mean difference (red. >75% vs. <75%)**	**P-value**	**Mean difference (red. >50% vs. <50%)**	**P-value**	**Mean difference (red. >25% vs. <25%)**	**P-value**
Arauca	-	-	-1.774 (4.803 vs. 3.029)	0.404	1.186 (3.451 vs. 4.637)	0.303
Armenia	3.637 (0.350 vs. 3.987)	**<0.001**	5.579 (0.116 vs. 5.695)	**<0.001**	91.876 (3.061 vs. 94.937)	**0.038**
Barranquilla	2.887 (0.249 vs. 3.137)	**<0.001**	-0.920 (5.191 vs. 4.271)	0.501	37.150 (2.429 vs. 39.579)	0.177
Bogotá, D.C.	1.387 (0.721 vs. 2.108)	**0.005**	0.623 (3.056 vs. 3.680)	0.470	-	-
Bucaramanga	3.680 (0.104 vs. 3.784)	**<0.001**	5.099 (2.766 vs. 7.865)	**<0.001**	-	-
Cali	2.256 (0.744 vs. 2.999)	**<0.001**	3.312 (0.762 vs. 4.074)	**<0.001**	31.042 (2.553 vs. 33.594)	**<0.001**
Cartagena de Indias	2.060 (1.252 vs. 3.312)	**0.005**	6.827 (2.348 vs. 9.176)	**<0.001**	-	-
Florencia	-34.299 (38.822 vs. 4.523)	0.469	-8.223 (14.061 vs. 5.838)	0.106	6.554 (6.276 vs. 12.830)	**0.008**
Ibagué	4.372 (0.231 vs. 4.603)	**<0.001**	5.804 (0.610 vs. 6.414)	**<0.001**	75.753 (3.715 vs. 79.468)	**<0.001**
Manizales	3.559 (0.168 vs. 3.727)	**<0.001**	9.097 (0.490 vs. 9.587)	**<0.001**	-	-
Medellín	3.508 (0.222 vs. 3.730)	**<0.001**	3.706 (3.049 vs. 6.755)	**0.007**	26.195 (3.176 vs. 29.371)	**0.003**
Mocoa	-	-	NA-	NA	0.073 (5.891 vs. 5.964)	0.982
Montería	2.079 (0.132 vs. 2.211)	**<0.001**	-1.953 (4.714 vs. 2.761)	0.280	6.035 (2.197 vs. 8.232)	**0.002**
Neiva	4.130 (0.457 vs. 4.587)	**<0.001**	9.167 (0.930 vs. 10.097)	**<0.001**	34.401 (3.880 vs. 38.281)	**0.013**
Pasto	3.403 (0.191 vs. 3.594)	**<0.001**	3.229 (4.168 vs. 7.396)	**0.035**	45.855 (3.051 vs. 48.906)	0.079
Pereira	3.121 (0.259 vs. 3.380)	**<0.001**	4.019 (0.309 vs. 4.327)	**<0.001**	45.529 (2.686 vs. 48.215)	**0.010**
Popayán	2.481 (0.614 vs. 3.096)	**<0.001**	4.384 (1.953 vs. 6.337)	**<0.001**	24.426 (2.970 vs. 27.396)	**0.002**
Quibdó	-	-	-3.046 (5.481 vs. 2.435)	**0.044**	4.004 (2.275 vs. 6.279)	**0.005**
Riohacha	1.702 (0.165 vs. 1.867)	**<0.001**	1.524 (2.302 vs. 3.826)	**0.049**	18.493 (1.671 vs. 20.165)	**<0.001**
San Andrés	-4.207 (8.737 vs. 4.530)	0.456	-2.354 (7.176 vs. 4.822)	0.268	-	-
San José de Cúcuta	2.098 (0.251 vs. 2.349)	**<0.001**	1.347 (2.755 vs. 4.102)	0.224	-	-
Santa Marta	1.482 (0.681 vs. 2.163)	**<0.001**	1.654 (2.607 vs. 4.261)	**0.030**	-	-
Sincelejo	-	-	-5.505 (8.065 vs. 2.559)	**0.018**	4.051 (2.912 vs. 6.963)	**<0.001**
Tunja	3.468 (0.465 vs. 3.933)	**<0.001**	8.886 (2.093 vs. 10.978)	**<0.001**	-	-
Valledupar	2.392 (0.291 vs. 2.683)	**<0.001**	2.423 (3.188 vs. 5.610)	**0.016**	12.160 (2.511 vs. 14.671)	**0.003**
Villavicencio	2.545 (0.458 vs. 3.003)	**<0.001**	3.155 (1.662 vs. 4.817)	**0.004**	15.994 (2.682 vs. 18.676)	**0.001**
Yopal	1.885 (0.657 vs. 2.541)	**<0.001**	3.820 (0.476 vs. 4.296)	**<0.001**	66.530 (2.223 vs. 68.753)	0.132

Note: Negative mean differences are increases in Covid-19 cases with more restrictions during the time-periods.

Supplementary Table 3. Differences in symptomatic Covid-19 case rates during the time with reductions of mobility trends in retail stores, at different cutoff values (25%, 50% and 75%) of weekly mobility reduction, in the imputed dataset.

**City**	**Reduction >75%**		**Reduction>50%**		**Reduction >25%**	
	**Mean difference (red. >75% vs. <75%)**	**P-value**	**Mean difference (red. >50% vs. <50%)**	**P-value**	**Mean difference (red. >25% vs. <25%)**	**P-value**
Arauca	NA	NA	-2.041 (4.803 vs. 2.762)	0.345	0.077 (4.059 vs. 4.135)	0.95
Florencia	-34.282 (38.822 vs. 4.540)	0.469	-8.126 (13.964 vs. 5.838)	0.071	6.558 (6.272 vs. 12.830)	**0.008**
Quibdo	NA	NA	-2.499 (4.791 vs. 2.292)	0.042	4.217 (2.062 vs. 6.279)	**0.004**
Riohacha	1.708 (0.165 vs. 1.873)	**<0.001**	1.155 (2.671 vs. 3.826)	0.106	18.442 (1.722 vs. 20.165)	**<0.001**
San Andres	-1.295 (5.825 vs. 4.530)	0.658	-0.913 (5.734 vs. 4.822)	0.588	NA	NA

Note: Only five cities had missing data: Florencia (5%), Quibdo (5%), Riohacha (7%), San Andres (7%), and Arauca (35%). Negative mean differences are increases in Covid-19 cases with more restrictions during the time-periods.

Supplementary Figure 1. (A) Forest plot of the meta-analysis of mean coefficients for the ARIMA model with reductions in retail mobility as regressor in each city of Colombia; (B) Covid-19 weekly case rate, below and above the 50% cutoff of reductions in mobility, per city; and (C) crude and adjusted mortality rates of Covid-19 in cities of Colombia.

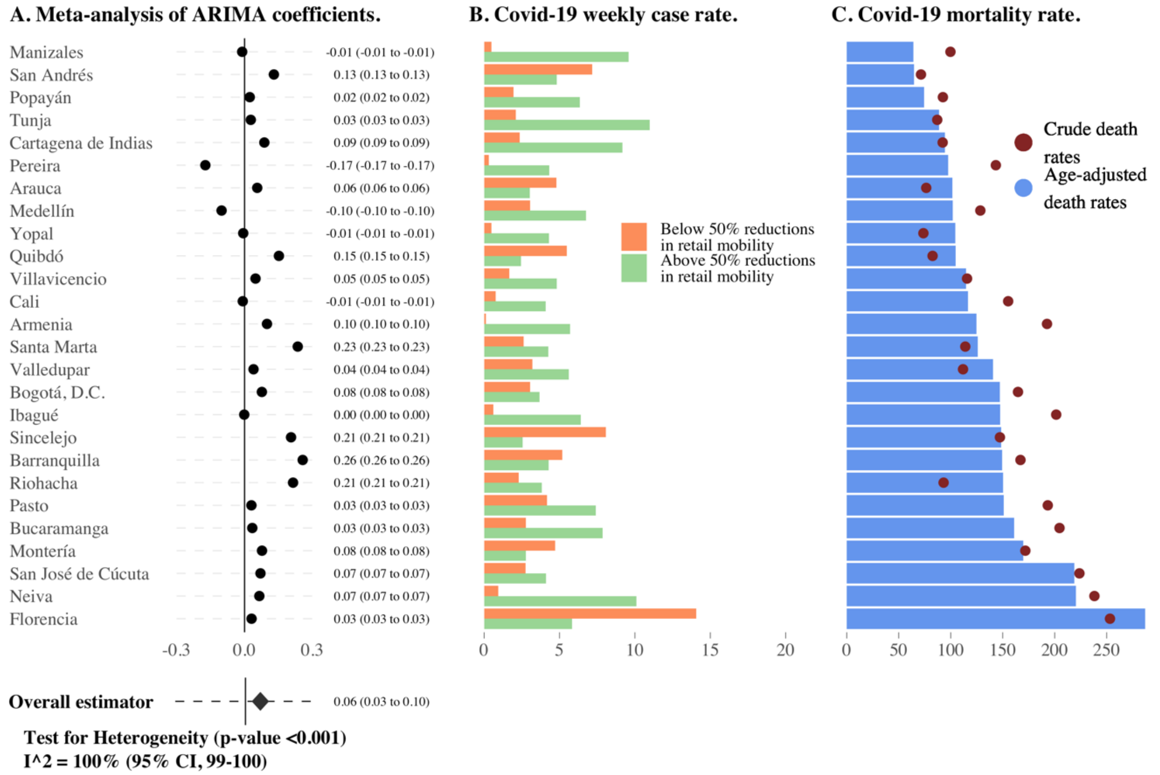

Note: We standardize the rates using 10-year age-groups in each city, using the United Nations population distribution as reference. The meta-analysis in (A) resulted in a mean coefficient of 0.0637 (95% confidence interval [CI], 0.027-0.101; P-value, <0.001). I^2 heterogeneity of 100%, and Q test for heterogeneity with a P-value <0.001. Panels (B) and (C) show the case and death rates per 100,000 population, respectively.

Supplementary Figure 2. Forest plot of the meta-analysis of mean coefficients for the ARIMA model with reductions in retail mobility as regressor in each city of Colombia, in a sensitivity analysis using data between January 3rd and February 6th of 2021.

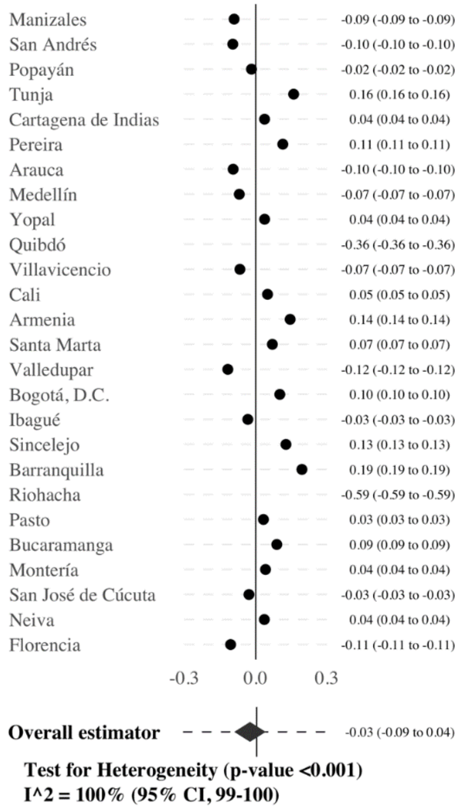

Supplementary Figure 3. Time trends of trends in mobility of retail stores in 27 cities of Colombia.

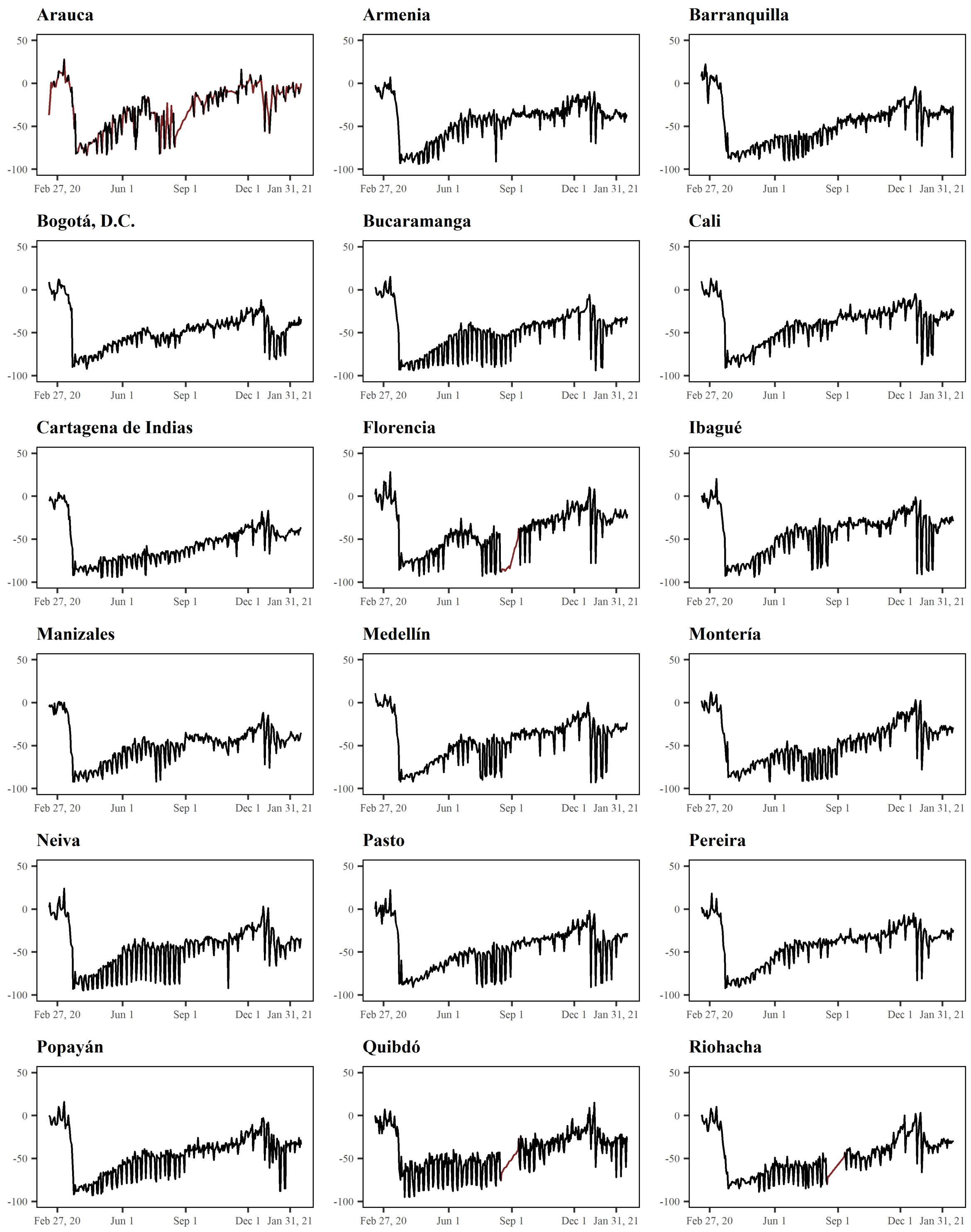

Supplementary Figure 3 (continued). Time trends of trends in mobility of retail stores in 27 cities of Colombia.

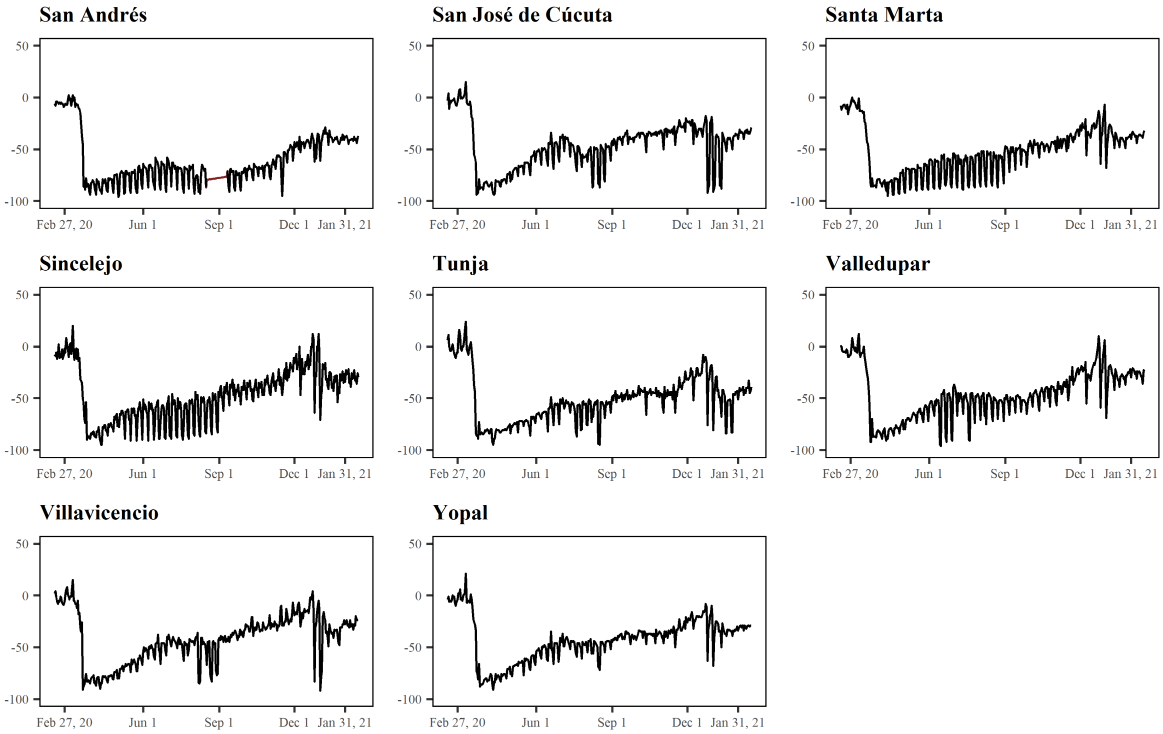

Note: Imputed time-series in red.

Supplementary Figure 4. Autocorrelation plots for the Arima models.

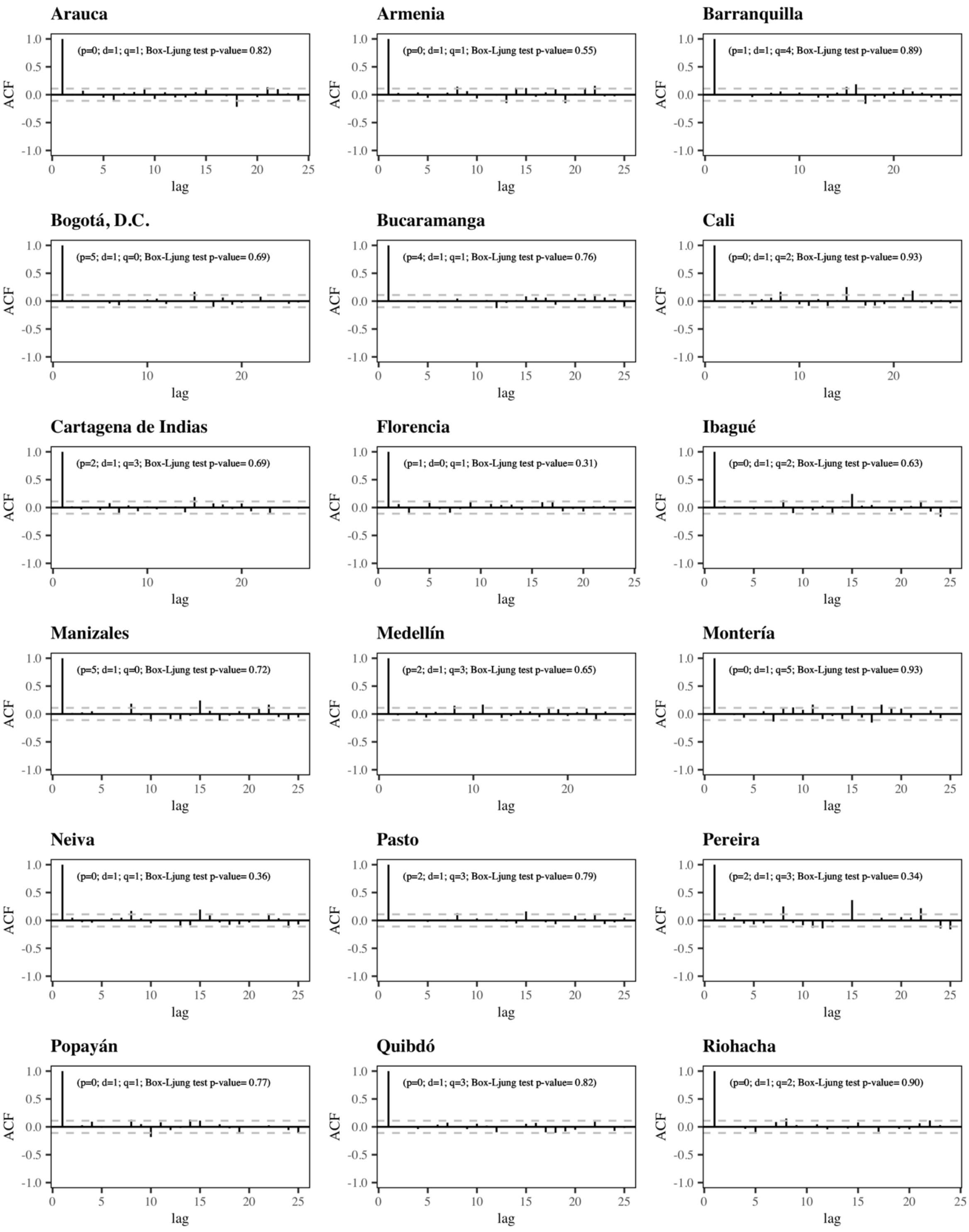

Supplementary Figure 4 (continued). Autocorrelation plots for the Arima models.

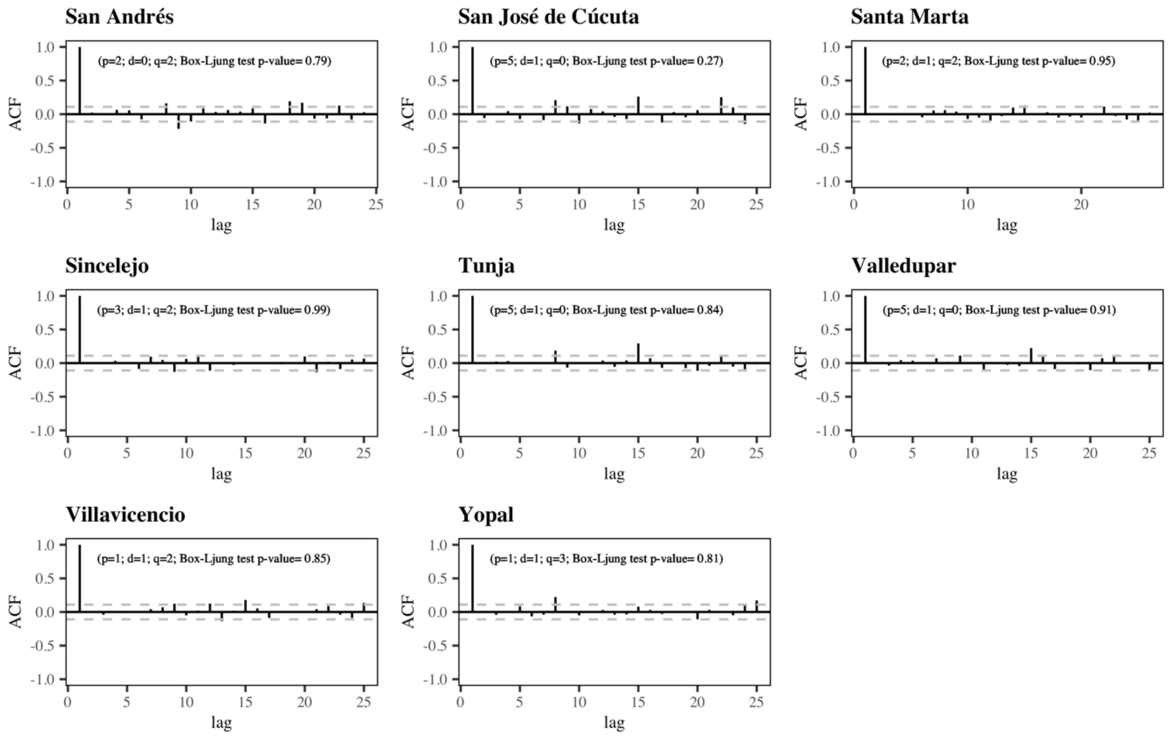

Supplement: ihab064_Supplemental_File [file ihab064_supplemental_file.pdf]
